# Effect of carbohydrate–protein supplementation on endurance training adaptations

**DOI:** 10.1007/s00421-020-04450-1

**Published:** 2020-08-05

**Authors:** Abdullah F. Alghannam, Iain Templeman, Joel E. Thomas, Dawid Jedrzejewski, Samuel Griffiths, Joseph Lemon, Thomas Byers, Sue Reeves, Javier T. Gonzalez, Dylan Thompson, James Bilzon, Kostas Tsintzas, James A. Betts

**Affiliations:** 1 Lifestyle and Health Research Center, Health Sciences Research Center, Princess Nourah Bint Abdulrahman University, Riyadh, 84428 Saudi Arabia; 2 Department for Health, University of Bath, Bath, BA2 7AY UK; 3 Department of Life Sciences, University of Roehampton, London, SW15 4JD UK; 4 School of Life Sciences, University of Nottingham, Queen’s Medical Centre, Nottingham, NG7 2UH UK

**Keywords:** Sucrose, Amino acids, Post-exercise nutrition, Recovery, Running

## Abstract

**Purpose:**

To examine the influence of post-exercise protein feeding upon the adaptive response to endurance exercise training.

**Methods:**

In a randomised parallel group design, 25 healthy men and women completed 6 weeks of endurance exercise training by running on a treadmill for 30–60 min at 70–75% maximal oxygen uptake (*V*O_2max_) 4 times/week. Participants ingested 1.6 g per kilogram of body mass (g kg BM^−1^) of carbohydrate (CHO) or an isocaloric carbohydrate–protein solution (CHO-P; 0.8 g carbohydrate kg BM^−1^ + 0.8 g protein kg BM^−1^) immediately and 1 h post-exercise. Expired gas, blood and muscle biopsy samples were taken at baseline and follow-up.

**Results:**

Exercise training improved *V*O_2max_ in both groups (*p* ≤ 0.001), but this increment was not different between groups either in absolute terms or relative to body mass (0.2 ± 0.2 L min^−1^ and 3.0 ± 2 mL kg^−1^ min^−1^, respectively). No change occurred in plasma albumin concentration from baseline to follow-up with CHO-P (4.18 ± 0.18 to 4.23 ± 0.17 g dL^−1^) or CHO (4.17 ± 0.17 to 4.12 ± 0.22 g dL^−1^; interaction: *p* > 0.05). Mechanistic target of rapamycin (mTOR) gene expression was up-regulated in CHO-P (+ 46%; *p* = 0.025) relative to CHO (+ 4%) following exercise training.

**Conclusion:**

Post-exercise protein supplementation up-regulated the expression of mTOR in skeletal muscle over 6 weeks of endurance exercise training. However, the magnitude of improvement in *V*O_2max_ was similar between groups.

## Introduction

Improved maximal oxygen uptake ($\dot{V}{\text{O}}_{2\max }$) is a hallmark adaptation to endurance exercise training, and is implicated as a key measure for human health and exercise performance (Jones and Carter 2000; Strasser and Burtscher 2018). Higher $\dot{V}{\text{O}}_{2\max }$ is linked to the reduction in all-cause mortality risk (Imboden et al. 2019), while also being regarded as the best indicator for endurance performance (Basset and Boulay 2000). Improvements in $\dot{V}{\text{O}}_{2\max }$ with endurance training involves an interplay between various organ systems: from cardiovascular adaptations to improve oxygen delivery (Convertino et al. 1980; Ekblom et al. 1968; Montero et al. 2015; Saltin et al. 1969; Schmidt et al. 1988), to skeletal muscle to improve oxidative capacity (Fritzen et al. 2019; Holloszy 1967; Hoppeler et al. 1985; Perry et al. 2010). Incorporating time-efficient strategies to maximise exercise-induced adaptations, including $\dot{V}{\text{O}}_{2\max }$, is, therefore, viable to favourably confer the desired health and performance outcomes.

Nutrient availability impacts the acute training response to endurance exercise (Coffey et al. 2011; Harber et al. 2010; Lunn et al. 2012), and can modulate chronic adaptations to physical training when applied repeatedly (Hawley et al. 2011). Both exercise and protein ingestion affect muscle protein kinetics (Breen et al. 2011; Lunn et al. 2012; Phillips et al. 1997), resulting in muscle remodelling contributing to training adaptations if sustained over weeks or months (Coffey et al. 2011; Farup et al. 2014; Morton et al. 2018; Perry et al. 2010). The “short-term” effects of protein ingestion on skeletal muscle adaptive response to an endurance exercise bout has been the focus of many investigations (Abou Sawan et al. 2018; Breen et al. 2011; Harber et al. 2010; Howarth et al. 2009; Lunn et al. 2012; Rowlands et al. 2015). Yet, the accumulation of these acute effects into chronic adaptive responses may be a more detectable and practically valuable outcome.

Limited information is available regarding the role protein supplementation during “long-term” (> 4 weeks) endurance training adaptations. The evidence at present is conflicting in terms of cardiovascular adaptations, with some (Ferguson-Stegall et al. 2011; Knuiman et al. 2019; Robinson et al. 2011), but not all (Jonvik et al. 2019) favouring post-exercise protein ingestion in potentiating exercise training improvements in $\dot{V}{\text{O}}_{2\max }$. Similarly, improved markers of mitochondrial adaptations (such as mitochondrial enzyme activity, PGC1-alpha content and mitochondrial DNA levels) following endurance training plus protein feeding have not been consistently reported (Ferguson-Stegall et al. 2011; Knuiman et al. 2019; Robinson et al. 2011). Inclusion of other potentially active ingredients such as caffeine and flavonoids (Ferguson-Stegall et al. 2011); selection of a control group not matched for macronutrient/energy content (Okazaki et al. 2009; Roberson et al. 2018); lack of double blinding (Ferguson-Stegall et al. 2011; Roberson et al. 2018); and insufficient sample size (Roberson et al. 2018; Robinson et al. 2011) make it difficult to conclude on the role of protein feeding in endurance training adaptations. Only two running-based studies were conducted, which did not measure $\dot{V}{\text{O}}_{2\max }$ changes in response to exercise training (Roberson et al. 2018), or utilised a different modality (i.e. cycling) in assessing $\dot{V}{\text{O}}_{2\max }$ (Robinson et al. 2011). It remains to be established whether protein supplementation enhances the adaptive response to running-based endurance training in recreationally active healthy young individuals.

We examined the effects of post-exercise whey protein ingestion relative to an isocaloric carbohydrate control on the cardiovascular and intra-muscular transcriptional adaptations to 6 weeks of running-based endurance training in recreationally active healthy young individuals. We hypothesised that carbohydrate–protein co-ingestion would increase $\dot{V}{\text{O}}_{2\max }$, alongside a differential expression of the key genes related to endurance training adaptation.

## Materials and methods

### Experimental design

The full experimental protocol for this trial was logged with a clinical trials register (ISRCTN27312291) and was published in advance (Alghannam et al. 2014). The primary outcomes were cardiovascular ($\dot{V}{\text{O}}_{2\max }$ and plasma albumin concentration) and intra-muscular (i.e. selected genes involved in cellular adaptive processes related to exercise/nutrition) responses to endurance training, and whether carbohydrate–protein co-ingestion could facilitate these adaptive responses. In a randomised investigator–participant double-blind parallel group design, participants were assigned (stratified for sex) to a group receiving a supplement containing carbohydrate (CHO trial) or carbohydrate plus protein (CHO-P trial). A total of 26 exercise training sessions were, therefore, prescribed for each participant (4 sessions per week). Mean adherence to exercise training (using the exercise logs and gym-based electronic system) was 96% (SD 4%). Consequently, participants completed 25 ± 1 and 25 ± 1 exercise training sessions in both CHO-P and CHO groups, respectively.

### Participants

Twenty-three healthy men and two healthy women participated in the study. The procedures and potential risks were explained to all participants both verbally and in writing prior to obtaining their written informed consent to take part, and administration of medical health questionnaires to ensure the absence of any risks associated with the nature of the study. This study was approved by the National Health Service (NHS) South West 3 Research Ethics Committee (13/SW/0239).

**Fig. 1:**
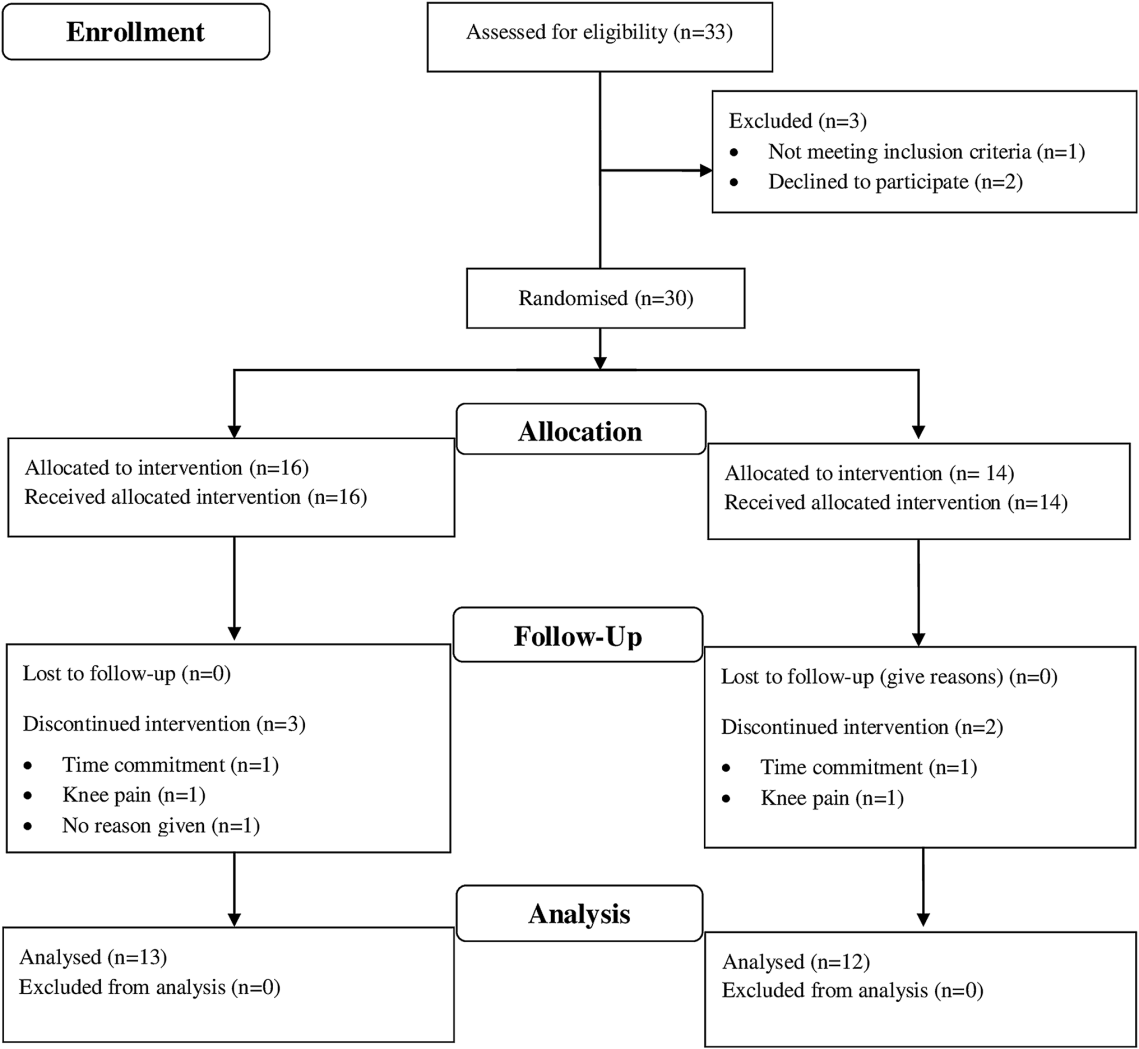
Participant recruitment and flow throughout the protocol. *CHO* carbohydrate group, *CHO-P* carbohydrate–protein group

Randomisation effectively generated two equivalent groups according to the good agreement between variables at baseline (Table 1). Thirty-three participants were initially recruited to take part in the study (Fig. 1) but three participants did not meet the eligibility criteria and were excluded from the outset. Furthermore, five participants withdrew from the study, two of whom sustained musculoskeletal injuries unrelated to the exercise training. Another 2 individuals reported time commitments as a reason for withdrawing from the study, whilst a single individual did not provide any information for withdrawal; thus 25 participants completed the study.

**Table 1:** Participant characteristics, including physiological parameters at baseline and follow-up

	All participants (*n* = 25)		CHO-P group (*n* = 13)		CHO group (*n* = 12)	
	Baseline	Follow-up	Baseline	Follow-up	Baseline	Follow-up
Age (years)	20 ± 2	–	20 ± 2	–	20 ± 2	–
Speed at 70% $\dot{V}{\text{O}}_{2\max }$ (km h^−1^)	10.2 ± 1.0	10.7 ± 0.9	10.0 ± 1.0	10.6 ± 0.9	10.4 ± 0.9	10.9 ± 0.9
Speed at 75% $\dot{V}{\text{O}}_{2\max }$ (km h^−1^)	11.0 ± 1.0	11.6 ± 1.0	10.8 ± 1.1	11.4 ± 1.0	11.2 ± 1.0	11.8 ± 0.9
Height (cm)	179 ± 10	–	179 ± 10	–	179 ± 10	–
Body mass (kg)	76.3 ± 12	75.4 ± 10	77.7 ± 13	76.0 ± 11	73.9 ± 9	74.0 ± 8
Body fat (%)	16.3 ± 4.5	15.9 ± 3.6	15.8 ± 5.2	15.4 ± 3.9	17.0 ± 3.8	16.4 ± 3.4
Body mass index (kg m^2^)	23.8 ± 2.8	23.6 ± 2.3	24.3 ± 3.4	23.8 ± 2.7	23.1 ± 2.0	23.1 ± 1.7
Resting heart rate (beats min^−1^)	61 ± 7	56 ± 9*	63 ± 6	57 ± 8*	59 ± 7	54 ± 10*
Maximum heart rate (beats min^−1^)	203 ± 10	197 ± 9*	200 ± 12	195 ± 10*	206 ± 7	199 ± 7*
Resting metabolic rate (kJ min^−1^)	7721 ± 259	7733 ± 225	7686 ± 322	7849 ± 238	7757 ± 301	7607 ± 301
Urine osmolality (mOsm kg^−1^)	519 ± 347	592 ± 281	523 ± 349	584 ± 291	516 ± 359	600 ± 282

Values are mean ± SD

*Significant time effect from baseline to follow-up (*p* < 0.05)

### Nutritional supplements

Participants in the CHO group received 1.6 g per kilogram of body mass (g kg BM^−1^) of carbohydrate (sucrose), whilst the CHO-P received an isocaloric carbohydrate (sucrose; 0.8 g kg BM^−1^) plus protein (whey protein hydrolysate; 0.8 g kg BM^−1^) supplement. Both treatments were divided into two boluses that were ingested immediately post-exercise and 1 h later. All supplements were provided in a sachet form and instructions for solution preparation were provided to participants to achieve a volume for ingestion of 10 mL kg BM^−1^ (8% solution). This precise amount of CHO was chosen to sufficiently refuel our participants. An example from one of our participants (male; 69 kg), running at 10.9 km h^−1^ (75% $\dot{V}{\text{O}}_{2\max }$) expends approximately 13.6 kcal min^−1^. The oxidation of CHO would be approximately 1.78 g min^−1^. This means that participant would oxidise 107 g CHO with a 60 min treadmill run at this given intensity. Thus, justifying why we provided a post-exercise recovery supplement in the CHO trial of 2 × 0.8 g kg BM^−1^ (total of ≈ 110 g). The amount of protein ingested (0.4 g kg BM^−1^ × 2 doses within an hour) is also on the upper range of what is required to maximally stimulate muscle protein synthesis. At that dose, any differences in anabolic responses between different protein types are diminished. Indeed, dietary protein intakes between 25 and 35 g per meal have been shown to maximally stimulate muscle protein synthesis in humans (Moore et al. 2015; Witard et al. 2014). For the above-mentioned reasons, the precise form of protein ingested (e.g. whey versus casein or soy) is not likely to be of huge importance in the context of this study. Nonetheless, plasma insulin response and branched-chain amino acids (BCAA) concentration increase to greater extent with ingestion of whey rather than casein in its intact form (Reitelseder et al. 2011). Moreover, the ingestion of whey protein hydrolysate exhibits larger circulatory increases in BCAA, essential amino acids than soy or casein protein in its isolated forms (Tang et al. 2009). An extension to these findings is that the ingestion of a protein hydrolysate (as used in this study) results in greater digestion and absorption compared with its intact protein, resulting in more rapid increase in circulating amino acid concentrations (Koopman et al. 2009). These findings are central to our choice of this protein source, given that increases in insulin and essential amino acids are potent modulators of plasma albumin (De Feo et al. 1992) and skeletal muscle protein synthesis (Fujita et al. 2007; Tipton et al. 1999), respectively.

In relation to the volume of fluid consumed, we used data obtained from a cohort of participants in our laboratory with similar characteristics (age 22 years; body mass 72.5 kg; exercise task: treadmill running at 70% $\dot{V}{\text{O}}_{2\max }$). The calculated sweat loss was 1.1 L; the sweat rate was 0.80 L h^−1^ (based on ad libitum water consumption, which was ≈ 0.4 L). Thus, the fluid provided post-exercise in this study was 2 × 10 mL kg BM^−1^ (i.e. ≈ 1.45 L), and so approximates 100–150% of body mass lost (1.1–1.65 L in this case), which falls within the fluid provided post-exercise in this study.

Participants in both groups were required not to consume any foods outside the prescribed supplements during the designated supplement provision periods (until 2 h immediately post-exercise). Adherence to supplement intake, time of ingestion and avoidance of any caloric intake outside the scope of the prescribed supplements were confirmed through a checklist, which was provided throughout the exercise training period for each participant. Full information concerning the nutritional treatments has been published previously with the registered protocol for this trial (Alghannam et al. 2014).

### Baseline testing

**Fig. 2:**
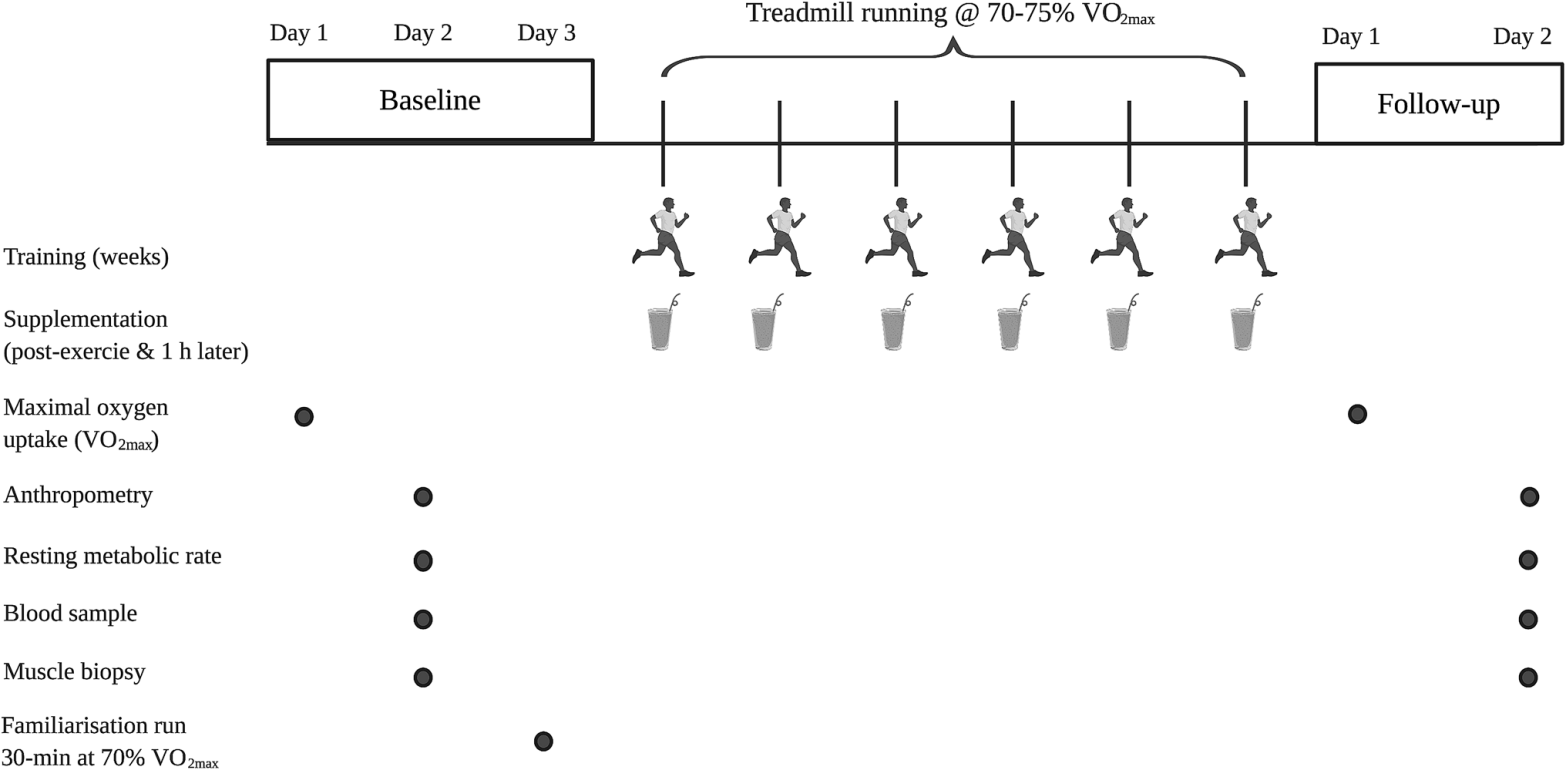
Schematic representation of the experimental protocol

Participants attended the laboratory on three separate occasions before commencing exercise training, and in two occasions post-intervention in accordance with the schematic representation illustrated in Fig. 2. We chose two visits to distinguish between resting data collection (following 48 h lifestyle standardisation) and minimise any potential discomfort between, for example, running until volitional exhaustion ($\dot{V}{\text{O}}_{2\max }$ testing) and taking a resting muscle biopsy sample. As our research question was not aimed at examining intra-muscular parameters during maximal exercise, we deemed it appropriate to minimise any discomfort by our participants by separating exhaustive exercise testing from resting measurements. The first visit included anthropometric assessment of stature (Wall-mounted Stadiometer, Seca, Hamburg, Germany) and body mass (Sliding Balance Scale Model 424, Weylux, UK), followed by a running economy (i.e. $\dot{V}{\text{O}}_{2}$ km^−1^) and $\dot{V}{\text{O}}_{2\max }$ test (Taylor et al. 1955) performed on a motorised treadmill (Ergo ELG70, Woodway, Weil am Rhein, Germany). The data acquired from these tests were subsequently used to calculate the treadmill speeds used during the trial procedures (i.e. speeds that elicit 60, 70 and 75% $\dot{V}{\text{O}}_{2\max }$) by linear regression (Excel 2010, Microsoft, Redmond, WA, USA). Ambient temperature, humidity and barometric pressure were monitored and recorded throughout the trials using a portable weather station (WS 6730; Technoline, Germany). The latter were used to record atmospheric pressure to allow for corrections to standard volumes during expired gas analysis.

The second pre-intervention visit was arranged within 7 days following the first, which was preceded by a 48 h standardisation of lifestyle (detailed below). Upon waking-up and prior to arrival at the laboratory, participants ingested 500 mL of water. Participants arrived at the laboratory at 08:00 ± 1 h following an overnight fast (≥ 10 h). A urine sample was collected on arrival to assess hydration via freezing point depression method using a cryoscopic osmometer (Advanced Instruments, Inc, Norwood, MA, USA), where adequate hydration was assumed for osmolality values ≤ 900 mOsm kg^−1^ (Shirreffs and Maughan 1998). Post-void nude body mass was measured (Weylux 424, Fereday & Sons Ltd., UK) before body fat percentage was determined using Bioelectrical Impedance Analysis (BIA; BC-543, Tanita, Tokyo, Japan). Thereafter, participants rested in a semi-supine medical bed for 5 min before expired gas samples (2 × 5-min Douglas bag collections) were taken to measure resting metabolic rate (RMR) along with resting heart rate via short-range telemetry (Polar FT2, Kempele, Finland) recorded at the final min of the expired gas collection period. Participants then remained in this semi-supine position in the medical bed while a 10 mL venous blood sample was then drawn through venepuncture (BD vacutainer, Plymouth, UK) from an antecubital vein. A 3–5-mm skin incision was then made from the lateral portion of the thigh (*vastus lateralis*) and an 80–100 mg muscle tissue sample was obtained using the needle biopsy technique under local anaesthetic (1% lidocaine; Hameln Pharmaceuticals Ltd., Brockworth, UK). Muscle biopsy samples were taken from the same leg at baseline and follow-up and separated by 2–3 cm, with the use of dominant/non-dominant legs counterbalanced between participants.

Following the two preliminary visits, participants performed two 30-min runs at 70% $\dot{V}{\text{O}}_{2\max }$ on a motorised treadmill (Ergo ELG70, Woodway, Weil am Rhein, Germany). This was intended to familiarise participants with the mode of exercise and verify the calculated relative intensity required during their prescribed exercise training.

### Exercise training

Participants underwent 6 weeks of progressive treadmill-based endurance exercise training. The duration of the entire protocol was 8 weeks and comprised (1) a baseline testing week with two exercise sessions; (2) the first, second and third weeks of exercise training at 70% $\dot{V}{\text{O}}_{2\max }$; (iii) the fourth, fifth and sixth weeks of exercise training at 75% $\dot{V}{\text{O}}_{2\max }$; and (4) a final week for follow-up testing. The duration of exercise sessions was progressively increased to 40, 50 and 60 min in week 1, weeks 2–3, and weeks 4–6 of the exercise training schedule, respectively. A 5-min warm-up at 60% $\dot{V}{\text{O}}_{2\max }$ preceded each exercise session, followed by treadmill running at a speed corresponding to 70% $\dot{V}{\text{O}}_{2\max }$. During the midpoint (training week 3), the speed was increased to elicit 75% $\dot{V}{\text{O}}_{2\max }$. Only water consumption was permitted during exercise sessions and this was consumed ad libitum. Once every fortnight, participants reported to the laboratory to be provided with the nutritional supplements for the subsequent 2-week exercise training block, with a total of three scheduled meetings throughout the exercise training.

### Follow-up testing

The follow-up procedures after the exercise training were identical to the initial two pre-intervention visits. Measurements of running economy and $\dot{V}{\text{O}}_{2\max }$ were taken at least one and at most 2 days following the final exercise training session. This was then followed by a 48-h standardisation of lifestyle (detailed below) before obtaining any measurement pertaining to the second follow-up laboratory visit.

### Expired gas sampling

The Douglas bag method using equipment (bags and respiratory valves) manufactured by Hans Rudolph (Shawnee, KS, USA) and supplied by Cranlea Human Performance Ltd (Birmingham, UK) was used for expired gas collection. The collected gas samples were analysed for relative expired fractions of oxygen and carbon dioxide using paramagnetic and infra-red analysers, respectively (Servomex, Crowborough, UK). The total volume of expired gas within the Douglas bag was subsequently measured by a dry gas meter (Harvard Apparatus, Kent, UK), with the temperature of expired gases being collected at the time of evacuation by a thermistor probe. Indirect calorimetry based on calculations (Frayn 1983) of oxygen consumption and carbon dioxide production from each bag was then used to obtain RMR in accordance with procedures recommended by The American Dietetic Association related for best practice in measuring RMR (Compher et al. 2006). The coefficient of variation for measurement of RMR in our laboratory using these procedures is 2.3%.

### Blood sampling

Venous blood samples were dispensed into 2 × 5 mL EDTA-treated tubes (Sarstedt, Leicester, UK). The first of these tubes was immediately analysed for haemoglobin concentrations (Sysmex SF-3000 Sysmex Ltd., Wymbush, UK) and haematocrit ratio (Hawksley, Lancing, UK). Mean corpuscular haemoglobin concentration was calculated by dividing haemoglobin concentration by haematocrit ratio. The remaining EDTA-treated blood was then centrifuged at 2000 × *g* for 10 min at 4 °C (Heraeus Primo R; Thermo Fisher Scientific, Loughborough, UK) for plasma extraction, then stored at − 80 °C for later analysis of plasma albumin concentration using an automated spectrophotometric analyser (RX Daytona, Randox, Crumlin, Ireland).

### Muscle tissue sample processing

Once removed from the *vastus lateralis*, each muscle sample was immediately immersed in liquid nitrogen and stored at − 80 °C pending analysis. Samples were then defrosted and transferred into an RNase-free conical tube (Corning, Ewloe, UK) containing 2 mL of Trizol (Invitrogen, Paisley, UK) and centrifuged at 4000 × *g* for 60 s at 4 °C before 400 µL of chloroform was added to the mixture. After shaking the mixture vigorously for 15 s, samples were incubated at room temperature for 3 min and then centrifuged at 4000 × *g* for 15 min at 4 °C. The aqueous phase was removed to a fresh conical tube and used for gene expression. The aqueous phase was mixed with 1500 µL of 100% ethanol before being loaded in an RNeasy mini column (Qiagen, Manchester, UK). Thereafter, the RNA was eluted using 25 μL of RNase-free water and 2 μL was then utilised for RNA quantitation using spectrophotometry (Spectrostar Nano, BMG Labtech, Ortenberg, Germany), with 400 ng of total RNA reverse transcribed using cDNA reverse transcription kit (Superscript III, Invitrogen, Paisley, UK).

### Quantitative real-time PCR

Taqman low-density custom array using Micro Fluidic cards (Applied Biosystems, Warrington, UK) was used for the relative quantification of expression of selected metabolic genes as previously described (Tsintzas et al. 2013). Each card allowed eight samples to be run in parallel against Taqman gene expression assay targets that were pre-loaded into each of the wells on the card. Briefly, 50 μL of Taqman Universal PCR master mix (Applied Biosystems, Warrington, UK) was added to 200 ng of cDNA in an Eppendorf RNAse-free tube. RNAse-free water was added to make the total volume of the reaction mixture up to 100 μL. The reaction mixture was mixed, centrifuged and loaded into one of the fill reservoir of the Micro Fluidic card. The cards were centrifuged (MULTIFUGE 3 SR; Heraeus, Thermo Fisher Scientific, Loughborough, UK) and run on a 7900HT Fast Real-Time PCR System (Applied Biosystems, Warrington, UK). Relative quantification of the genes of interest was performed using the comparative CT method. The average expression of two housekeeping genes [actin, alpha 1 (ACTA1) and hydroxymethylbilanesynthase (HMBS)] was used to normalise the data.

### Standardisation of lifestyle

Participants were asked to maintain their habitual dietary energy intake and activity levels (i.e. no substantial changes in caloric intake, dietary habits or physical activity levels outside the scope of the prescribed supplements and training sessions) over the course of the experiment. A weighed dietary record and an exercise activity log were completed by participants for 48 h before baseline testing and replicated prior to follow-up testing. These dietary records were analysed using nutritional analysis software (Nutritics LTD, Dublin, Ireland) and there were no differences between the CHO (mean ± SD; 10,092 ± 2117 kJ day^−1^; 51 ± 7% carbohydrate; 30 ± 8% fat; 19 ± 5% protein) and CHO-P (9699 ± 2448 kJ day^−1^; 51 ± 7% carbohydrate; 32 ± 7% fat; 17 ± 2% protein) groups. Participants also abstained from alcohol consumption and refrained from strenuous physical activity, with any light exercise recorded and matched during the 48-h period of standardisation of lifestyle for follow-up testing. Three 3-day dietary records were collected and analysed from participants at different intervals during the 6 weeks of exercise training, to provide a reflection of dietary intake habits between groups over the exercise training period.

Participants were instructed not to participate in any other structured exercise session over the period of the experiment and to ingest a meal (breakfast or lunch depending on the time of day) approximately 2 h prior to any exercise session and thereafter abstain from any caloric intake prior to the commencement of the training session. Moreover, an exercise log was provided to each participant to record the relevant information relating to each exercise session (e.g. time of day and total duration of session), which was then verified by an electronic monitoring system that requires participants to use a key card to enter and leave the gymnasium. The exercise logs were collected and cross-examined with the electronic monitoring system to verify adherence for each exercise session attendance, date and duration.

### Statistical analysis

Independent effects of treatment group (CHO vs CHO-P) and time (baseline vs follow-up) and interaction effects (group × time) were explored using a two-way linear mixed model with repeated measures on the time variable. Any data that require a single comparison of two means were assessed for normality using the Shapiro–Wilk test before using an independent *t* test or non-parametric equivalent (Mann–Whitney) to examine differences between treatment arms (e.g. magnitude of change in $\dot{V}{\text{O}}_{2\max }$ between treatments). A priori sample size estimation (G*power version 3.1.7, University Düsseldorf, Düsseldorf, Germany) was performed using previous cycling-based post-exercise nutrition and endurance training data (Ferguson-Stegall et al. 2011) and revealed that a total of 24 participants were required to achieve 80% power to detect a worthwhile increase in endurance capacity (i.e. $\dot{V}{\text{O}}_{2\max }$) of 5.3 mL kg^−1^ when ingesting carbohydrate–protein supplements vs a carbohydrate supplement, with a standard deviation of 3.3 mL kg^−1^ using a two-tailed *t* test at an alpha level of 0.05. Rolling recruitment of ≈ 30 participants was, therefore, conducted in consideration of an anticipated 15% dropout rate (Alghannam et al. 2014). Significance was set at *p* ≤ 0.05 and data are reported as the mean ± standard deviation (SD) unless stated otherwise.

## Results

### Participant characteristics

No group × time interactions were identified in any participant characteristics shown in Table 1 between groups. Moreover, no changes occurred from baseline to follow-up in anthropometric measures in either group. Resting and maximal heart rates were reduced following exercise training in both treatments (*p* < 0.01), with no difference in these responses between groups. Assessments of baseline resting metabolic rate were closely matched between the CHO-P and CHO groups (Table 1) and were stable within ≈ 155 kJ day^−1^ from baseline to follow-up, with no notable differences between groups at follow-up (243 kJ day^−1^). Similarly, the hydration status of participants assessed via urine osmolality measurements was similar across groups, with no differences between groups at baseline or follow-up.

### Dietary analysis

Dietary intake estimated by 3 × 3-day dietary records during exercise training did not reveal any differences between groups: CHO-P 9.3 ± 2.5 MJ day^−1^; 47 ± 6% carbohydrate; 30 ± 4% fat; 20 ± 5% protein; and CHO 9.9 ± 3.0 MJ day^−1^; 51 ± 6% carbohydrate; 29 ± 5% fat; 17 ± 2% protein (mean ± SD), excluding the intake of nutritional supplements, which were isocaloric. Overall, the dietary intake excluding supplementation was not different during exercise training and the 48-h baseline dietary records.

Total energy intake with supplementation was 12.0 ± 2.9 MJ day^−1^ (62 ± 5% carbohydrate; 24 ± 4% fat; 14 ± 1% protein) in the CHO group and 11.4 ± 2.5 MJ day^−1^ (50 ± 5% carbohydrate; 25 ± 4% fat; 25 ± 5% protein) in the CHO-P group. Macronutrient intake relative to body mass was 6.1 ± 1.3 g carbohydrate kg BM^−1^ day^−1^; 1.1 ± 0.4 g fat kg BM^−1^ day^−1^; 1.4 ± 0.3 g protein kg BM^−1^ day^−1^ in the CHO group and 4.6 ± 1.1 g carbohydrate kg BM^−1^ day^−1^; 1.1 ± 0.4 g fat kg BM^−1^ day^−1^; 2.3 ± 0.5 g protein kg BM^−1^ day^−1^ in CHO-P group. Thus, relative daily carbohydrate intake was higher in the CHO group (*p* = 0.016) and the amount of protein ingested was higher in the CHO-P group (*p* < 0.001).

### Maximal oxygen uptake

**Fig. 3:**
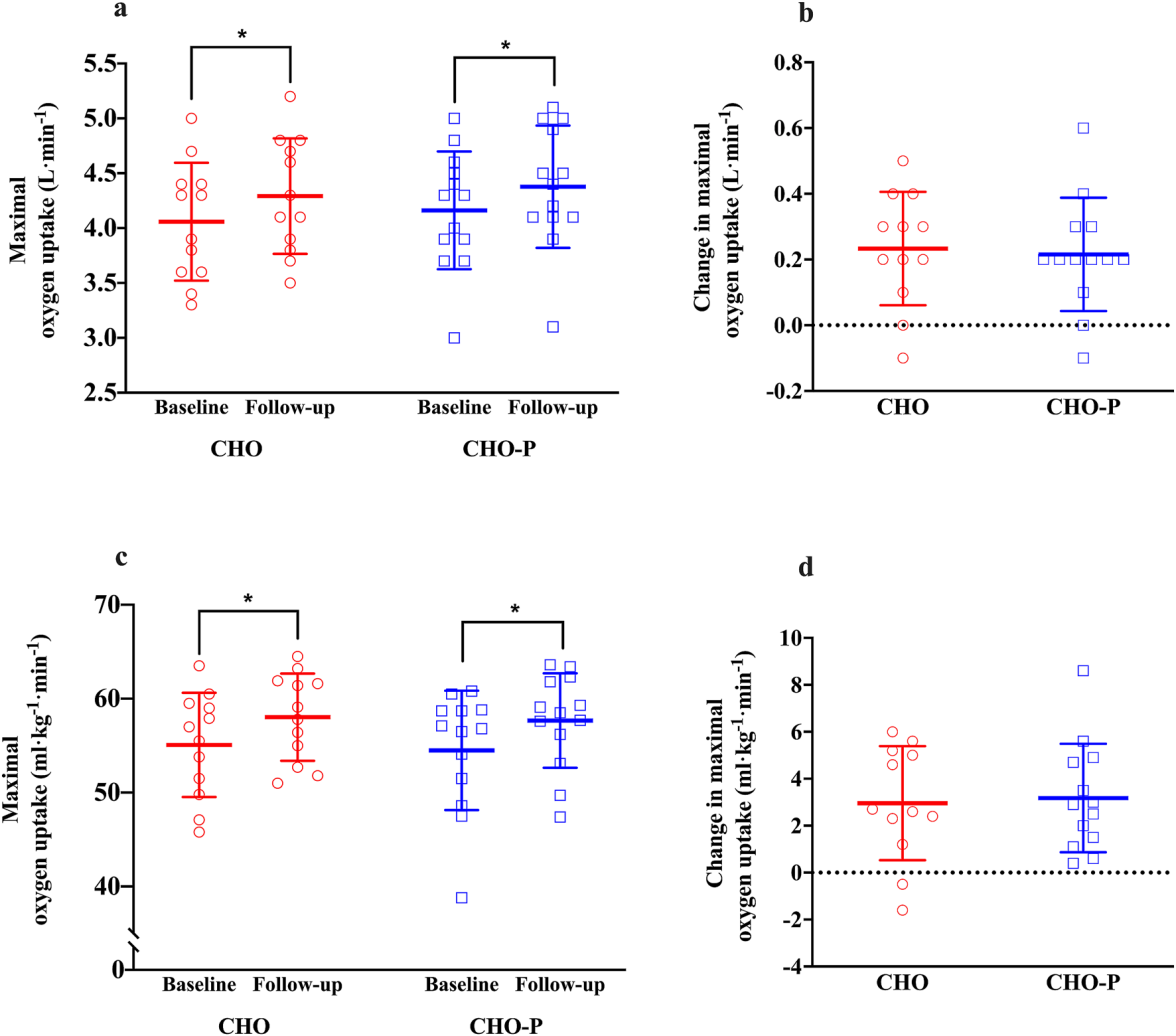
Maximal oxygen uptake ($\dot{V}{\text{O}}_{2\max }$)in CHO (*n* = 12; red coloured) and CHO-P (*n* = 13; blue coloured) groups. The open symbols represent individual scores; the horizontal and vertical lines represent mean ± standard deviation, respectively. **a** Absolute $\dot{V}{\text{O}}_{2\max }$ scores in L min^−1^ from baseline to follow-up in both groups; **b** change in $\dot{V}{\text{O}}_{2\max }$ in L min^−1^ from baseline to follow-up for both groups; **c** relative $\dot{V}{\text{O}}_{2\max }$ scores in mL kg^−1^ min^−1^ from baseline to follow-up in both groups; **d** change in $\dot{V}{\text{O}}_{2\max }$ in mL kg^−1^ min^−1^ from baseline to follow-up in both groups. *Significant main effect of time compared with baseline (*p* < 0.01). *CHO* carbohydrate, *CHO-P* carbohydrate–protein

Absolute and relative $\dot{V}{\text{O}}_{2\max }$ of the study population improved in response to 6 weeks of treadmill running by 0.2 ± 0.2 L min^−1^ and 3.0 ± 2 mL kg^−1^ min^−1^ (*p* < 0.001). The magnitude of improvement from baseline to follow-up in both absolute and relative $\dot{V}{\text{O}}_{2\max }$ was not different between groups (Fig. 3), with improvements from baseline of 5.3 ± 4% and 5.3 ± 4% in CHO-P and CHO treatments, respectively. The change in both absolute and relative $\dot{V}{\text{O}}_{2\max }$ was not different between groups, as reflected by no group × time interactions (*p* > 0.05; Fig. 3).

### Plasma volume and blood constituents

**Fig. 4:**
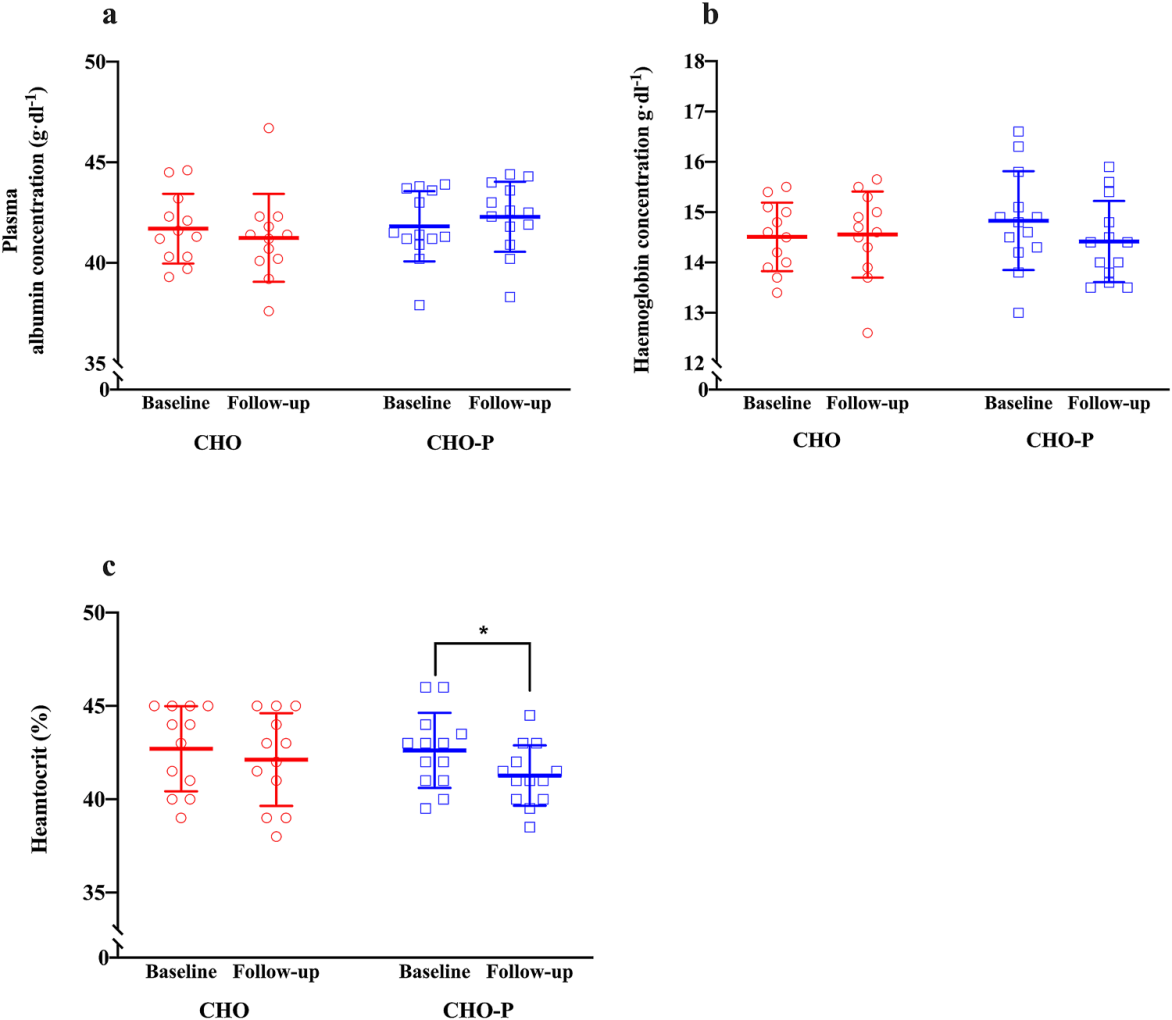
Haematological parameters collected during the study in CHO (*n* = 12; red coloured) and CHO-P (*n* = 13; blue coloured) groups. The open symbols represent individual scores; the horizontal and vertical lines represent mean ± standard deviation, respectively. **a** Plasma albumin concentrations in g dL^−1^ from baseline to follow-up in both groups; **b** haemoglobin concentrations in g dL^−1^ from baseline to follow-up in both groups; **c** haematocrit ratio (%) from baseline to follow-up in both groups. *Significant main effect of time compared with baseline (*p* < 0.01). *CHO* carbohydrate, *CHO-P* carbohydrate–protein

No group × time interaction was observed in plasma albumin concentration (*F* = 1.5; *p* = 0.23; Fig. 4). Haemoglobin concentration tended to be lower (− 2.7%) following exercise training in CHO-P group (time: *p* = 0.09; Fig. 4) with no group × time interaction (*F* = 1.99; *p* = 0.17). Haematocrit ratio was 3.1% lower following exercise training in the CHO-P group (time: *p* = 0.01; Fig. 4), and no group × time interaction (*F* = 1.6; *p* = 0.20). Mean corpuscular haemoglobin concentration did not change from baseline to follow-up (time: *p* = 0.23) in either the CHO group (34.0 ± 0.8 to 34.6 ± 1.9 g dL^−1^) or the CHO-P group (34.8 ± 1.1 to 34.9 ± 1.0 g dL^−1^), again with no group × time interaction (*F* = 0.68; *p* = 0.42).

### Intramuscular parameters

**Fig. 5:**
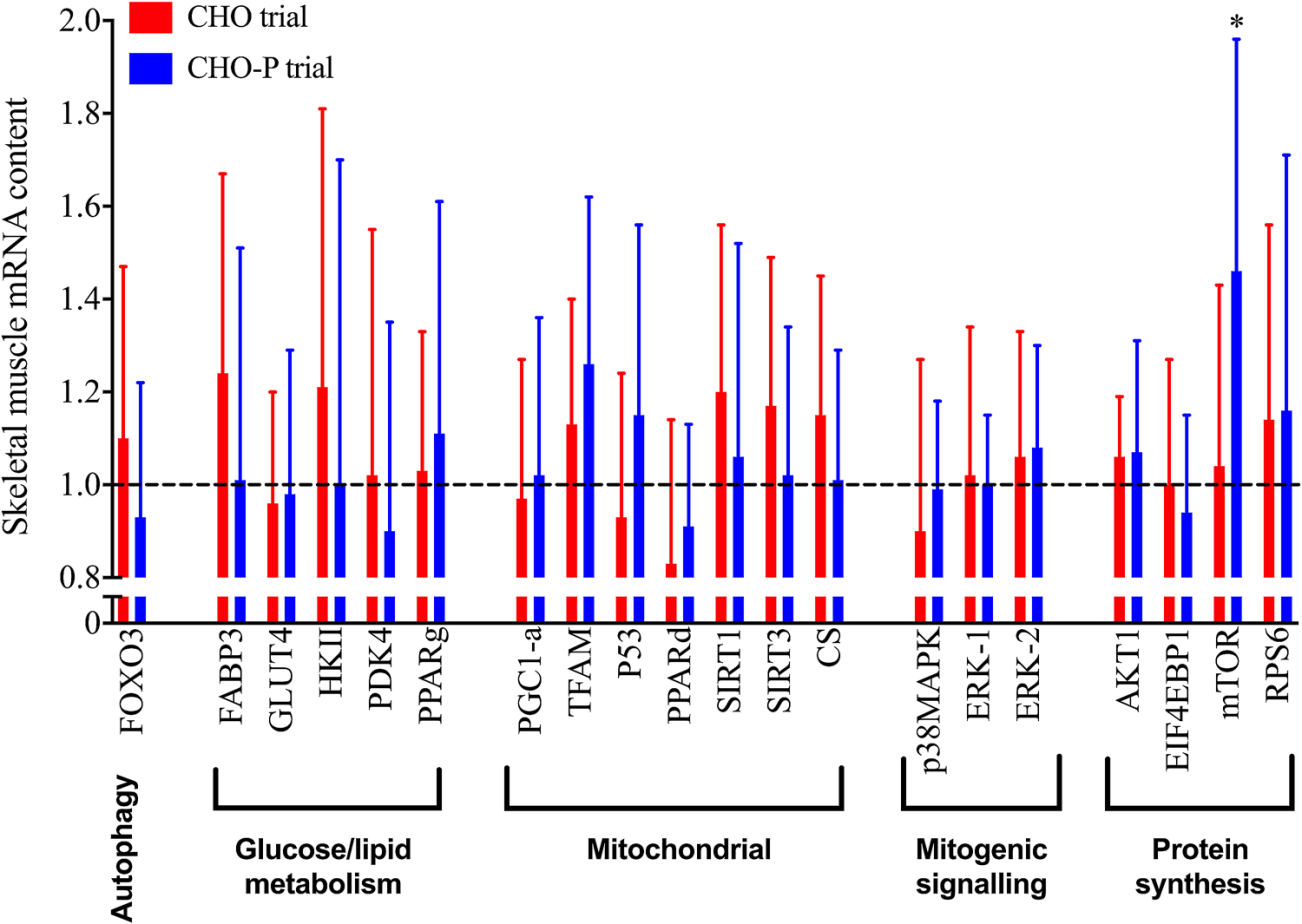
Relative gene expression represented as fold change from baseline for several key genes related to mitochondrial biogenesis, muscle protein synthesis and carbohydrate/lipid metabolism. Values are mean ± SD. *p < 0.05 baseline vs follow-up. *FOXO3* forkhead box O3, *FABP3* fatty-acid binding protein 3, *GLUT4* glucose transporter protein 4, *HKII* hexokinase II, *PDK4* pyruvate dehydrogenase kinase 4, *PPARg* peroxisome proliferator-activated receptor gamma, *PGC1-a* peroxisome proliferator-activated receptor gamma coactivator 1-alpha, *TFAM* mitochondrial transcription factor A, *P53* tumour suppressor p53, *PPARd* peroxisome proliferator-activated receptor delta, *SIRT1* NAD-dependent deacetylase sirtuin-1, *SIRT3* NAD-dependent deacetylase sirtuin-3, *CS* citrate synthase, *p38MAPK* P38 mitogen-activated protein kinase, *ERK1* mitogen-activated protein kinase 3, *ERK2* mitogen-activated protein kinase 1, *AKT1* AKT serine/threonine kinase 1, *EIF4EBP1* eukaryotic translation initiation factor 4E binding protein 1, *mTOR* mechanistic target of rapamycin, *RPS6* ribosomal protein S6

Figure 5 illustrates the change in skeletal muscle expression of key genes in related to endurance training adaptation in CHO-P (*n* = 8) and CHO (*n* = 7). The overall expression of mechanistic target of rapamycin (mTOR) was up-regulated in response to training (+ 27%; *p* = 0.05). The expression of mechanistic target of rapamycin (mTOR) was up-regulated from baseline to follow-up only the CHO-P group (+ 46%; *p* = 0.03). Mitochondrial transcription factor A (TFAM) gene expression was up-regulated in CHO-P group (26%) when compared with CHO (13%), albeit this did not reach statistical significance (*p* = 0.07). No differences in the change in expression of other measured metabolic genes (Fig. 5) were observed between CHO-P and CHO treatments (*p* > 0.05).

## Discussion

We report that completing 6 weeks of regular treadmill running was sufficient to increase $\dot{V}{\text{O}}_{2\max }$ but there were no differences in the magnitude of this improvement between the two treatment arms. However, post-exercise carbohydrate–protein co-ingestion up-regulated the expression of mTOR in skeletal muscle following training relative to an energy-matched post-exercise carbohydrate-only solution.

The magnitude of improvements in $\dot{V}{\text{O}}_{2\max }$ in response to endurance training in the present study is in line with others (Egan et al. 2013; Fritzen et al. 2019; Hoppeler et al. 1985; Milanovic et al. 2015). We did not find protein-mediated improvements in $\dot{V}{\text{O}}_{2\max }$ in the present study; concurrent with some (Jonvik et al. 2019; Okazaki et al. 2009), but not all (Ferguson-Stegall et al. 2011; Knuiman et al. 2019; Robinson et al. 2011) investigations. The magnitude of $\dot{V}{\text{O}}_{2\max }$ response depends on other factors than the intensity and frequency of training including: genetic predisposition, initial fitness status, age and testing modality (Basset and Boulay 2000; Bouchard et al. 1999; Bouchard and Rankinen 2001; Caputo et al. 2003; Jones and Carter 2000). The baseline $\dot{V}{\text{O}}_{2\max }$ in our cohort was 9–50% higher when compared with other studies, suggesting diminishing gains in this parameter with endurance exercise training compared with lower baseline $\dot{V}{\text{O}}_{2\max }$ scores (Bouchard and Rankinen 2001), particularly when considering the greater magnitude of improvement in $\dot{V}{\text{O}}_{2\max }$ in untrained young (Ferguson-Stegall et al. 2011) and sedentary older individuals (Robinson et al. 2011) with endurance training and post-exercise protein ingestion. We measured $\dot{V}{\text{O}}_{2\max }$ during treadmill running, which yields ≈ 10–15% higher scores than cycling (Caputo et al. 2003); a testing modality used by all others to determine $\dot{V}{\text{O}}_{2\max }$ (Ferguson-Stegall et al. 2011; Jonvik et al. 2019; Knuiman et al. 2019; Okazaki et al. 2009; Robinson et al. 2011). It is reasonable to infer that $\dot{V}{\text{O}}_{2\max }$ trainability is a single, inherently heterogenic indicator of endurance training adaptations, and other physiological parameters should supplement this measure to assess overall endurance capacity or performance (e.g. time to exhaustion or time trial performance testing).

Haematological adaptations with endurance exercise training play an important role in mediating improvements in $\dot{V}{\text{O}}_{2\max }$ (Bonne et al. 2014; Montero et al. 2015; Schmidt and Prommer 2010). Changes in maximal cardiac output following 6 weeks of endurance training are ascribed to an expansion in blood volume (Bonne et al. 2014), and both plasma and erythrocyte volume expansions are implicated in this phenomenon (Sawka et al. 2000). Despite that we did not measure plasma and erythrocyte volumes in this experiment, there was a significant reduction (− 3.1%) in haematocrit in the CHO-P group. This has been observed in the previous literature in relation to high-intensity interval training and in that case was taken to reflect hypervolaemia (Eigendorf et al. 2018). However, the absence of such decreases in haematocrit in the CHO group does not rule out the possibility of similarly increased blood volume when carbohydrate was ingested without protein, especially considering that mean corpuscular haemoglobin concentration did not respond differently between treatments. Given that intramuscular markers of aerobic capacity do not appear to explain the observed increase in $\dot{V}{\text{O}}_{2\max }$, it seems reasonable to suggest that the training adaptation in both groups may be attributable to increased hypervolaemia. Hypervolemia is modulated by plasma protein content (Gillen et al. 1991), in particular plasma albumin (Convertino et al. 1980). Hepatically derived plasma protein synthesis is stimulated by the acute ingestion or infusion of amino acids (Caso et al. 2007; De Feo et al. 1992). Despite providing protein in amounts (0.4 g kg BM^−1^ h^−1^) sufficient to maximise albumin synthetic response (Moore et al. 2009), we did not observe a significant increase in plasma albumin concentration in the CHO-P group (Fig. 4). However, Okazaki et al. (2009) showed that post-exercise protein feeding with endurance exercise training improved plasma albumin content and stroke volume in older individuals (Okazaki et al. 2009), and this was supported by improved $\dot{V}{\text{O}}_{2\max }$ in a similar population (Robinson et al. 2011). Older individuals may not consume adequate daily amounts of protein (Landi et al. 2016); thus post-exercise protein ingestion enhanced albumin content compared with the placebo group (Okazaki et al. 2009). Dietary records from our study show that daily protein intake was adequate in both groups (2.3 g protein kg^−1^ day^−1^ vs 1.4 g protein kg^−1^ day^−1^ in CHO-P and CHO, respectively), which may explain the similarities in albumin concentrations between groups. Noteworthy is that we did not directly measure plasma volume to enable measurements of albumin content rather than concentration; an important implication given the possibility that hypervolemia may have occurred at a higher magnitude in CHO-P group. It remains possible that post-exercise protein feeding may accentuate haematologic and cardiovascular adaptation, and further investigations are required to clarify albumin-mediated effects with protein feeding on endurance training adaptations in healthy young individuals.

Mitochondrial biogenesis is an important adaptation to increase the oxidative capacity of the contracting muscle (Fritzen et al. 2019; Holloszy 1967; Perry et al. 2010), and requires a coordinated expression of nuclear genes encoding mitochondrial proteins and genes encoded in the mitochondrial genome (Holloszy 2008; Hood 2001). Transcription regulators (TFAM) and co-activators (PGC1-alpha) are important in regulating mitochondrial biogenesis and adaptation to endurance exercise (Olesen et al. 2010), and have been shown to increase with 2–8 weeks of endurance training (Egan et al. 2013; Popov et al. 2018). Translational expression of TFAM protein content is associated with its transient increases in mRNA expression after each exercise bout (Popov et al. 2018). Interestingly, we saw an up-regulation in the expression of TFAM mRNA following 6 weeks of endurance training with protein supplementation, although this was not statistically significant (*p* = 0.07). To our knowledge, only one study reported 2 weeks of post-exercise protein supplementation may enhance (PGC1-alpha) mRNA expression measured 6 h following an acute exercise bout (Hill et al. 2013). However, it is difficult to ascertain whether these effects were related to chronic or acute supplementation, given that these measurements followed acute post-exercise ingestion with varied macronutrient intakes (carbohydrate vs carbohydrate–protein). We provide additional insight into gene-specific temporal patterns of induction of mRNA expression of mitochondrial biogenesis in response to 6-week treadmill running and following 48 h of lifestyle standardisation to exclude any acute effects on transcriptional changes. Our data supports the notion of gene and time course specificity of the regulation of gene expression in response to exercise and nutrition. Thus, mitochondrial transcript level adjustment and subsequent protein content may require ≥ 6 weeks to detect measurable increases in mitochondrial content in response to endurance training (Hood 2001; Popov et al. 2018).

Protein intake after endurance exercise modulates signalling pathways involved in translation initiation and transcriptome implied in repair and remodelling of structural and contractile elements of skeletal muscle (Abou Sawan et al. 2018), which ultimately mediates important adaptive responses to endurance exercise (Hawley et al. 2011). A central cellular regulator of translation initiation and subsequent protein synthesis is the mechanistic target of rapamycin (mTOR), which is stimulated by both muscular contractions (Drummond et al. 2009) and protein feeding (Atherton and Smith 2012). mTOR translation initiation and protein content are up-regulated following both acute and chronic muscular contractions (Coffey et al. 2011; Edgett et al. 2013), possibly by a reduced myostatin-mediated activation of the mTOR pathway (Konopka and Harber 2014). mTOR also translocates to the cell periphery following endurance exercise, and this colocalisation is associated with an increase in myofibrillar muscle protein synthesis (Abou Sawan et al. 2018), sensitising it to receive amino acid substrates (Song et al. 2017). This regulatory process is important in regulating myofibrillar mRNA translation and subsequent protein synthesis in human skeletal muscle (Song et al. 2017). We observed an up-regulation in the expression of mTOR (*p* = 0.03) following endurance training in the CHO-P group, suggesting myofibrillar protein synthesis is primarily sensitive to nutrient provision (Abou Sawan et al. 2018). Considerable muscle hypertrophy occurs with endurance training (Konopka and Harber 2014) and post-exercise protein ingestion seems to accentuate this response (Knuiman et al. 2019). Ingesting protein following endurance exercise substantially elevates myofibrillar muscle protein synthesis (Abou Sawan et al. 2018; Rowlands et al. 2015). This may partly explain improvements in $\dot{V}{\text{O}}_{2\max }$, given its positive correlation with changes in lean body mass, at least in cycling (Knuiman et al. 2019). Although we did not see an accentuated improvement in $\dot{V}{\text{O}}_{2\max }$, the potential benefit of protein feeding in other adaptive responses to endurance training such as tissue repair remodelling would support its central role in post-exercise recovery nutrition.

We provided ≈ 30 g of whey protein post-exercise and 1 h later, sufficient to maximise muscle protein synthesis (Moore et al. 2009) and consequently drive adaptations to endurance training. Our protein feeding strategy provided greater amounts of daily protein intake than other studies showing that protein increases the magnitude of gains form endurance exercise training (Ferguson-Stegall et al. 2011; Knuiman et al. 2019; Robinson et al. 2011). Ingesting the protein supplement in the current study increased their daily protein intake to 2.3 g kg^−1^ day^−1^, while it was 1.4 g kg^−1^ day^−1^ in the CHO group. The recommended daily protein intake for endurance activities falls within a range of 1.2–1.7 g kg^−1^ day^−1^ (Tarnopolsky 2004). Given that the non-protein supplemented group in our study met the aforementioned daily protein intakes, it can be argued that ingesting 1.4 g protein kg^−1^ day^−1^ is sufficient to meet the protein requirements to support endurance training in our cohort. Protein requirements are reflective of the endurance exercise volume (Moore et al. 2014), and the current evidence recommends protein intakes of 1.8–2.1 g kg^−1^ day^−1^ for endurance trained individuals (Bandegan et al. 2019; Kato et al. 2016). Our participants ran ≈ 40 km per week (5 h per week) in a moderate–high-intensity exercise, and it likely that daily protein recommended intake for such training frequency/volume is ≈ 1.8 g kg^−1^ day^−1^ (Kato et al. 2016), particularly when considering that previously untrained individuals may require more protein to optimise muscle protein synthesis and lean body mass accretion (Simmons et al. 2016). Despite the substantial increase in daily protein intake through protein supplementation in CHO-P group, no additional benefits occurred in cardiorespiratory fitness. The higher total daily protein intake in the CHO group may have potentially masked the effect of protein supplementation in our investigation.

To our knowledge, this is the first randomised double-blind controlled trial exploring the effect of post-exercise protein feeding on prolonged running-based endurance exercise training in healthy young individuals. The experiment was designed to isolate the effects of added protein; hence, the control was carbohydrate only matched for energy content, yet a group with no post-exercise supplementation at all would have answered additional research questions. Thus, it is not possible to directly assess the effect of training stimulus/nutrient intake per se on the outcome measures obtained, only the specific added effect of protein as per our hypothesis. In addition, whilst participants’ hydration status was controlled via standardised fluid intake prior to blood sampling (as verified by urine analysis) and posture was also fixed for all blood draws (15-min semi-supine rest), interpretation of the haematological parameters reported here should be balanced against the potential for small differences in hydration and/or body position to mask small treatment effects. Finally, the current experiment explored relative change in the basal expression of selected genes associated with endurance training adaptation, and thus the findings related to intra-muscular adaptive response to endurance training plus post-exercise supplementation are only limited within the context of this measure and at the basal time point. It is conceivable that the magnitude of increase in transcriptional and mitochondrial proteins may have been magnified between the experimental groups, and that changes in mRNA expression following each bout of exercise could have been missed. Nevertheless, the current approach allowed us to explore the chronic effect of endurance training on the basal expression of key genes. Therefore, any changes in basal expression are likely to persist for longer, thereby having the potential to meaningfully influence protein translation.

In conclusion, post-exercise carbohydrate–protein ingestion up-regulated muscle mTOR expression following 6 weeks of treadmill running training, compared to an isocaloric carbohydrate-only solution. While exercise training improved maximal oxygen uptake, the magnitude of this effect was not different with the inclusion of protein in a post-exercise carbohydrate solution when compared to an isocaloric carbohydrate-only supplement.

## Abbreviations

ACTA1: Actin, alpha 1 housekeeping gene
BCAA: Branched chain amino acids
BIA: Bioelectrical impedance analysis
BM: Body mass
cDNA: Complementary deoxyribonucleic acid
CT method: Cycle threshold method
EDTA: Ethylenediaminetetraacetic acid
HMBS: Hydroxymethylbilane synthase housekeeping gene
mRNA: Messenger ribonucleic acid
mTOR: Mechanistic target of rapamycin
PCR: Polymerase chain reaction
PGC1-alpha: Peroxisome proliferator-activated receptor gamma coactivator 1-alpha
RMR: Resting metabolic rate
RNA: Ribonucleic acid
SD: Standard deviation
TFAM: Mitochondrial transcription factor A
$\dot{V}{\text{O}}_{2} \cdot {\text{km}}^{ - 1}$: Oxygen uptake per kilometre
$\dot{V}{\text{O}}_{2\max }$: Maximal oxygen uptake
